# Temporal and spatial patterns and determinants of traditional villages in Henan Province

**DOI:** 10.1371/journal.pone.0350290

**Published:** 2026-05-28

**Authors:** Man Zhang, Jiabin Wu, Yu Liu

**Affiliations:** 1 School of Management, Fujian University of Technology, Fuzhou, Fujian Province, China; 2 School of Civil Engineering and Transportation, Anyang Institute of Technology, Anyang, Henan Province, China; 3 School of Flight, Anyang Institute of Technology, Anyang, Henan Province, China; Xi’an University of Architecture and Technology, CHINA

## Abstract

Traditional villages are important carriers of China’s cultural heritage and reflect long-term interactions among history, environment, and human settlement. This study examines 1,035 officially recognized traditional villages in Henan Province to identify their spatiotemporal distribution patterns and associated factors. Using ArcGIS-based spatial analysis, GeoDetector, the spatial lag model, and historical literature review, we find that traditional villages show significant spatial clustering and clear regional inequality. High-density clusters occur in northern Henan (Anyang–Hebi), central Henan (Pingdingshan), and southern Henan (Xinyang), whereas eastern Henan remains sparsely distributed, partly due to the long-term impacts of Yellow River flooding. The evolution of village distribution can be divided into four major historical stages, and the dominant spatial orientation shifted from a southwest–northeast trend before the Ming period to a southwest–northeast trend thereafter. Descriptively, village concentration is associated with moderate elevation, proximity to water, lower GDP levels, and intermediate distance from county-level central cities. However, after spatial autocorrelation was controlled for, these variables were not statistically significant as independent predictors in the spatial lag model, suggesting that village distribution is shaped more by combined effects and spatial spillover than by single factors alone. These findings provide evidence for cluster-based heritage protection in Henan and offer broader lessons for historically layered inland regions facing rural transformation and urbanization pressures.

## 1. Introduction

Traditional villages, often described as ancient villages, function as living systems that preserve both tangible and intangible cultural heritage, including historical settlement forms, architectural practices, social organization, and local knowledge [1]. Rather than being relics, these villages actively reflect the production modes, lifestyles, and socio-economic structures of specific historical periods, making them valuable for examining long-term regional and national cultural evolution [2,3]. Their architectural forms and decorative traditions integrate functional designs with aesthetic expression, providing important reference materials for architecture, cultural studies, and related disciplines [4,5]. However, previous studies have shown that rapid industrialization and urbanization, together with rural population outmigration, land-use conversion, and the replacement of traditional buildings, have placed increasing pressure on traditional villages and contributed to the decline or disappearance of many traditional settlements in China in recent decades [6–8]. This trend highlights the urgent need for systematic research on the spatiotemporal patterns, driving mechanisms, and preservation risks of traditional villages to inform targeted conservation and sustainable development strategies [6].

Henan Province, located in China’s Central Plains and central to the agricultural civilization in the Yellow River Basin, contains traditional villages with distinct spatial and cultural characteristics from those of other regions [5,9,10]. As a long-term political and cultural center of the Central Plains, Henan’s historical capitals strongly shaped settlement distribution and continuity. Long-term agricultural development in the Central Plains further supported a stable village system, contributing to the persistence of traditional settlements [11]. Culturally, Henan integrates influences from Chu, Wu, Huaiyi, and agricultural traditions, a synthesis reflected in village layouts, architectural styles—such as Han-style “One-seal” courtyards and pit courtyards, and folk traditions, including temple fairs and agricultural rituals [1,9,12]. These features distinguish Henan’s traditional villages from the ethnic-symbiotic clusters found in Southwest China [13] and the defense-oriented coastal villages of Southeast China [14,15].

Spatially, traditional villages in Henan Province exhibit a pattern of overall dispersion and local aggregation. Most villages occupy gentle mid-elevation terrain between 100 and 300 meters and closely align with river corridors, with approximately 79.61% located within 2000 meters of rivers or lakes [9,16]. This distribution differs from the mountainous aggregation of villages in Yunnan [13] and the predominantly plain-based settlement patterns in Jiangnan villages [17]. Henan’s location within the Yellow River and Huaihe River basins helps explain this pattern, as villages historically balanced agricultural suitability with the need to reduce flooding exposure [5,11]. Simultaneously, Henan’s status as China’s most populous province creates distinctive pressures on traditional villages. Urbanization-driven population outflow has intensified village hollowing [6,7], weakened intergenerational transmission of intangible cultural heritage, and encouraged construction practices that disrupt historical architectural styles [9]. Despite these challenges, the province’s dense population base—historically foundational and currently sustained—continues to support cultural continuity and partially mitigate village decline, distinguishing Henan from sparsely populated regions in the upper reaches of the Yellow River Basin [5].

Research on traditional villages has expanded rapidly across multiple spatial scales and regions. National-level studies have mapped broad distribution patterns [6,10], whereas regional and local studies have focused on major river basins, such as the Yellow River [5] and Yangtze River [11], and on areas including Southwest China [13] and the Southeast Coast [14,18]. These studies consistently highlight the roles of natural conditions, particularly topography and water systems [5,9], as well as socio-economic factors, such as transportation infrastructure and policy interventions [8,16], in determining village distribution. However, despite Henan’s central position in Chinese agricultural and political history, systematic spatial analyses of traditional villages in the Central Plains remain limited [9,16].

Three major gaps remain in the existing literature on traditional villages. First, relatively few studies systematically link Henan’s historical role as an imperial political center and its long-standing population density to the formation and evolution of traditional villages. Consequently, prior research often underestimates the combined effects of political changes, agricultural expansion, and population mobility on settlement patterns in the region [11,12]. Second, many regional comparative studies overlook Henan’s unique dual river basin constraints and its culturally integrative character, which limits understanding of the spatial adaptation mechanisms that differentiate Henan from other regions [5,9]. Third, most studies fail to adequately integrate empirical spatial analysis with theoretical frameworks of rural settlement geography in the Central Plains, leading to weak theoretical explanations of regional heterogeneity [19–21]. For instance, research on the Yellow River Basin has emphasized natural environmental factors [5]. In contrast, the Henan context requires explicit consideration of historical institutional change and agricultural technological development, which are insufficiently addressed in current scholarship [1,16].

This study addresses these gaps by theoretically and empirically contributing to the study of traditional villages in China. Conceptually, this study treats the distribution of traditional villages as the outcome of three interrelated processes: environmental constraint, historical path dependence, and uneven urbanization. Environmental conditions such as topography and water systems define the basic suitability and risk structure of rural settlement. Historical political centrality, agricultural expansion, and migration generate path-dependent settlement persistence, causing earlier cores of habitation to continue shaping present-day village patterns. At the same time, urbanization does not affect all places equally; villages located in less-developed and moderately accessible areas may be more likely to retain traditional forms because they are buffered from intensive redevelopment while remaining socially and economically viable. From this perspective, “human–environment coordination” refers to the long-term adaptation of settlements to terrain and hydrology, whereas “regional heterogeneity” refers to the uneven ways these processes combine across different parts of Henan.

Theoretically, it advances the understanding of regional heterogeneity by quantitatively examining the spatiotemporal patterns and influencing factors of traditional villages in Henan province [11,16]. Methodically, the study applies ArcGIS-based spatial analysis techniques, such as kernel density estimation and nearest neighbor index, with geographic detector models to identify key drivers of village distribution and evolution [12,22]. By focusing on villages located within a historically significant political center and a densely populated area, the analysis strengthens the empirical foundation of the study of settlement geography in the Central Plains [1]. Furthermore, the study extends the theoretical framework of human-environment coordination by analyzing how traditional villages in Henan adapt to the combined constraints of dual river basins and cultural integration [11,15].

The findings of this study provide actionable guidance for the targeted protection and sustainable development of traditional villages in Henan, tackling issues such as the prioritization of formal declaration over effective management and the homogenization of cultural tourism development [7,8]. By identifying high-density village clusters in the northern, central, and southern regions, this research informs the planning of contiguous protection zones and prioritization strategies. The results also support cultural heritage activation by emphasizing multicultural integration—measurable via resource activation, communication innovation, and community benefits—while guiding rural revitalization policies that balance preservation with population retention [5,9,16]. These insights offer a reference framework for other historically significant and densely populated regions in China, enabling context-sensitive strategies for conserving traditional villages [11,12].

This study focuses on Henan’s traditional villages with three primary objectives: (1) characterizing their spatiotemporal distribution and evolutionary trajectories; (2) testing how environmental constraints, historical path dependence, and contemporary socioeconomic conditions jointly shape these patterns; and (3) deriving region-specific as well as transferable implications for heritage preservation under urbanization.

## 2. Materials and methods

### 2.1. Study area

Henan Province lies at the heart of China’s Central Plains, spanning the middle and lower reaches of the Yellow River (31°23′–36°22′ N, 110°21′–116°39′ E). It covers approximately 167,000 square kilometers and borders Shandong to the east, Hebei and Shanxi to the north, Shaanxi to the west, and Hubei and Anhui to the south. As a strategic crossroads linking northern, southern, eastern, and western China, Henan’s topography is dominated by plains, which account for 55.7% of the area, complemented by hills and mountains. The Yellow River and Huaihe River basins traverse the province, creating fertile agricultural land and hydrological conditions that historically supported dense human settlement [9,16].

The historical significance of Henan underpins its distinctive settlement patterns. Serving as the capital for 20 dynasties, the province contains seven ancient capitals—Luoyang, Kaifeng, Anyang, Zhengzhou, Xuchang, Luohe, and Shangqiu—that have left lasting imprints on regional villages [1]. For example, Anyang, the capital of the Shang Dynasty (1600–1046 BCE), preserves oracle bone inscriptions and royal mausoleums, reflecting early administrative and ritual organization. Luoyang served as the capital of 13 dynasties, including the Eastern Zhou, Eastern Han, Wei, Sui, and Tang, and evolved into a major cultural and diplomatic hub. Kaifeng, the capital of the Northern Song Dynasty (960–1127 CE), was one of the world’s largest cities at the time, with a population exceeding one million and thriving commerce that shaped surrounding rural settlement patterns [5,9]. The political centrality of these capitals fostered dense village networks that served as agricultural supply bases, handicraft workshops, laying the foundation for both the spatial distribution and cultural continuity of Henan’s traditional villages [18]. Agricultural supply drove the formation of pre-modern satellite clusters (later becoming the Anyang-Hebi and Pingdingshan hotspots); handicraft specialization ensured settlement continuity (reflected in stable central Henan kernel density); and socio-economic condition hubs mediated the directional shift from “Southwest-Northeast” to “Northwest-Southeast” (see Section 3.2).

Henan’s historical position as a cultural crossroads has fostered the integration of diverse cultural systems, including those of Chu, Wu, Huaiyi, and the Central Plains agricultural cultures. This integration is evident in village layouts, such as defensive courtyards designed in response to historical conflicts, and in architectural styles, such as Han-style “One-seal” courtyards and pit courtyards adapted to local environmental conditions. Local folk traditions, including temple fairs, agricultural rituals, and other intangible cultural heritage linked to ancient capitals, further reflect this cultural synthesis [1,9]. Unlike the ethnic-symbiotic villages of Southwest China or the coastal defense-oriented settlements of Southeast China, Henan’s traditional villages demonstrate both the continuity of Han civilization and long-term adaptation to an agrarian economy [16,18].

Henan remains one of China’s most populous provinces, with a permanent population exceeding 98 million in 2023, of which approximately 42% (around 41 million) reside in rural areas. The province’s rural population density, approximately 245 individuals per square kilometer, contrasts sharply with the sparsely populated upper reaches of the Yellow River Basin and the more urbanized lower reaches [5,16]. This dense rural population has shaped human-land interactions: it has preserved traditional agricultural practices and cultural heritage, while also intensifying pressures, such as land scarcity, population outflow, and “village hollowing” amid urbanization [6,7]. Notably, high rural population concentrations in northern (Anyang-Hebi), central (Pingdingshan), and southern (Xinyang) Henan closely correspond with clusters of traditional villages, illustrating the persistent interplay between demographic patterns and settlement distribution [9].

The intersection of Henan’s historical role as a political capital, its rich cultural integration, and its dense rural population has shaped traditional villages with distinctive regional characteristics that differentiate them from settlements in other river basins or regions. This study focuses on Henan’s traditional villages, as documented in national catalogues, to analyze their spatiotemporal evolution within a framework of long-term historical accumulation and dynamic human-land interactions.

### 2.2. Data sources and period classification logic

A dataset of 1,035 traditional villages was compiled, including both national-level Chinese traditional villages and those specifically located in Henan Province. National-level data, encompassing six batches and totaling 275 villages, was sourced from the Chinese Traditional Village Network (www.chuantongcunluo.com), while Henan-specific data, also organized in six batches with 760 villages, were obtained from the Henan Provincial Department of Housing and Urban-Rural Development (https://hnjs.henan.gov.cn/bsjdcx/). Gaode Map API (v 2.0) was used to accurately geocode village locations and determine the geometric centers of administrative boundaries. Ambiguous locations were cross-validated with Baidu Maps and Tencent Maps, adopting consensus coordinates as the final values. To assess the reliability of the coordinate data, a random sample of 10% of the villages (n = 104) was manually verified using Baidu Maps. For each sampled village, the recorded coordinates were compared with the location identified through place-name matching and map image inspection. The average positional deviation between the recorded and verified locations was less than 100 m, indicating acceptable accuracy for the spatial analyses conducted in this study (S1 Appendix). The corresponding attribute data were integrated using ArcGIS 10.2. The administrative boundary vector data of the study area was obtained from the 1:250,000 vector map information database provided by the national basic geographic information center (http://www.ngcc.cn/ngcc/). Water system data was obtained from the resource and environmental science and data center of the Chinese Academy of Sciences (http://www.resdc.cn/). Using Euclidean distance in ArcGIS 10.2, calculate the distance between the village and the water system. Administrative boundary points and river vector data were acquired from the National Basic Geographic Database (scale 1:4 million) and transformed into the GCS WGS 1984 coordinate system, then standardized to the WGS 1984 Web Mercator Auxiliary Sphere projection format to maintain spatial compatibility.

Village evolution was classified into four critical periods (Table 1, S1 Appendix) by integrating multi-source data to determine founding or formation times and establishing standardized chronological classification rules. Temporal data were cross-validated across three sources. Primary sources included official records, such as the Henan Provincial Traditional Village Protection List (2023) and county-level gazetteers (e.g., Anyang and Xinyang Municipal Records), which provide founding years, clan histories, and significant historical events, such as migrations or temple constructions. Secondary sources comprised village cultural heritage surveys and protection archives from the Henan Provincial Department of Housing and Urban-Rural Development, which offer architectural age data, genealogical records, and oral histories from village elders. Finally, supplementary sources included academic research on Central Plains rural settlements and archeological reports, including Yangshao and Longshan cultural site studies, to resolve ambiguities in village chronologies.

**Table 1:** Chronological classification of traditional villages in Henan Province.

Temporal Period	Time Range	Classification Criteria
Pre-Qin Period	Before 221 BCE	Villages associated with Neolithic/Bronze Age cultural sites (e.g., Pei Ligang, Yangshao) or recorded in pre-Qin historical texts (e.g., Shiji).
Tang, Song, and Yuan Periods	618–1368 CE	Villages with confirmed Tang/Song/Yuan architectural remains (e.g., stone pagodas, ancient wells) or official records of settlement establishment in dynastic histories.
Ming and Qing Periods	1368–1912 CE	Villages with clear genealogical records, ancestral hall construction dates (1368–1912), or county gazetteer entries confirming formation during this era.
Post-Qing Period	1912–1945 CE	Villages founded after the fall of the Qing Dynasty, confirmed by post-1912 migration records or new settlement documents (e.g., anti-Japanese base area archives).

### 2.3. Research methods

#### 2.3.1. Nearest neighbor index.

The Nearest Neighbor Index (NNI) was used to quantify the spatial configuration of traditional villages. The NNI evaluates the distribution pattern of point features by comparing the observed average nearest neighbor distance of villages to the expected distance under a hypothetical random distribution. It provides a clear measure of clustering, randomness, or uniformity in village locations. Mathematically, the NNI is defined as follows:


$$\begin{array}{c} R=\frac{r_{\text{1}}}{r_{E}}=\text{2}\sqrt{D}\times r_{\text{1}} \end{array}$$ (1)

$$\begin{array}{c} r_{E}=\frac{\text{1}}{\text{2}\sqrt{D}} \end{array}$$ (2)

where R is the nearest neighbor index, $r_{\text{1}}$ is the observed average nearest neighbor distance of the villages, and $r_{E}$ is the expected distance assuming a random distribution, and D is the point density, calculated as the number of villages per unit area. The NNI values are interpreted following standard conventions: R less than 1 indicates clustering, a value equal to 1 suggests a random distribution, and a value greater than 1 indicates a uniform distribution [23].

#### 2.3.2. Thiessen polygon model.

The Thiessen polygon model was applied to analyze the spatial relationship among traditional villages in Henan Province. This model partitions a spatial plane into polygons, with each polygon surrounding a point such that any location within the polygon is closer to its central point than any other. By examining the area and variability of these polygons, we can evaluate the proximity and spatial distribution patterns of the point set. Tessellated polygon analysis was employed in this study to quantify these distribution characteristics. The calculations are defined as follows:


$$\begin{array}{c} R=\sqrt{\sum \frac{{\left(S_{i}-S\right)}^{\text{2}}}{n}}(i=\text{1},\text{2},\ldots ,\text{n}) \end{array}$$ (3)

$$\begin{array}{c} C_{v}=R/\text{S} \end{array}$$ (4)

Here, $C_{v}$ is the coefficient of variation; $C_{v}$ is the standard deviation of the polygon area; $S_{i}$ is the area of the *i*-th polygon; *S* is the average area of the polygons, and *n* is the number of polygons. A low $C_{v}$ value indicates that the points are uniformly distributed, indicating minimal variability in the polygonal areas. Conversely, a high $C_{v}$ value suggests clustered point distributions, with significant variation in polygon sizes [24].

#### 2.3.3. Geographical concentration index.

The Geographical Concentration Index was used to quantify the degree of clustering of traditional villages across Henan Province. This index evaluates how unevenly a phenomenon, such as population, economic activity, or settlement, is distributed within a defined geographical area. In this study, we first counted the number of traditional villages in each of Henan’s 158 county-level administrative districts. We then normalized these counts by the total number of villages in the province to calculate the index, which reflects the spatial concentration of villages across the administrative regions. The Geographical Concentration Index is defined as follows:


$$\begin{array}{c} G=\text{1}\text{0}\text{0}\times \sqrt{\sum_{i=\text{1}}^{n}{\left(\frac{x_{i}}{T}\right)}^{\text{2}}} \end{array}$$ (5)

*G* represents the geographical concentration index, *x*_*i*_ is the number of villages in district *i*, *T* is the total number of villages across all districts, and n is the number of county-level districts (*n* = 158). The index ranges from 0 to 100, with higher values indicating a stronger concentration of villages and lower values indicating a more even or dispersed distribution [18].

#### 2.3.4. The imbalance index.

The Imbalance Index (*S*) was used to quantify the variation in the spatial aggregation of traditional villages across Henan’s county-level administrative districts. This index measures the degree to which village distribution is uneven among districts, providing insight into areas with concentrated or dispersed settlements. The imbalance index is calculated as follows:


$$\begin{array}{c} S=\frac{\sum_{i=\text{1}}^{n}Y_{i}-\text{5}\text{0}(n+\text{1})}{\text{1}\text{0}\text{0}n-\text{5}\text{0}(n+\text{1})} \end{array}$$ (6)

Where *S* represents the imbalance index, *n* = 158 is the total number of counties and districts in Henan Province, and *Y*_*i*_ denotes the cumulative percentage of villages in the leading counties when the districts are ranked in descending order by village count. The index ranges from 0 to 1: *S* = 0 indicates a perfectly uniform distribution of villages across all districts, whereas *S* = 1 indicates that all villages are concentrated in a single district. Therefore, higher *S* values indicate greater spatial imbalance and stronger aggregation of traditional villages [9].

#### 2.3.5. Standard deviation ellipse.

The standard deviation ellipse was used to quantitatively analyze the spatial characteristics and directional trends of traditional villages in Henan Province. This spatial statistical tool calculates three key parameters: the center of gravity, the azimuth angle, and the two principal axes: major and minor. The major axis represents the dominant direction of spatial extension, while the minor axis reflects the dispersion perpendicular to this direction. The shape of the ellipse is further described by the oblateness ratio, which indicates the degree of directional concentration: a high oblateness ratio indicates significant data aggregation in a specific direction, whereas a low ratio, where the ellipse approximates a circle, indicates weak directionality and increased dispersion of points [12].

#### 2.3.6. Kernel density estimation method.

We applied the kernel density estimation (KDE) method, a non-parametric approach, to quantify the spatial concentration of traditional villages in Henan Province. KDE estimates the density of point features across a given area, with higher density values indicating a greater spatial frequency of villages and stronger spatial interactions among them. Each village is treated as an independent event, and the density at any location reflects the combined influence of nearby villages. The KDE function is expressed as follows:


$$\begin{array}{c} f_{n}(x)=\frac{\text{1}}{nh}\sum_{i=\text{1}}^{n}k(\frac{x-x_{i}}{h}) \end{array}$$ (7)

Here, $f_{n}(x)$ represents the estimated density at location x, n is the total number of villages, h is the bandwidth controlling the spatial extent of influence for each point, k is the kernel weight function, and $x-x_{i}$ measures the distance between the estimation point and the village [25].

**Fig 1:**
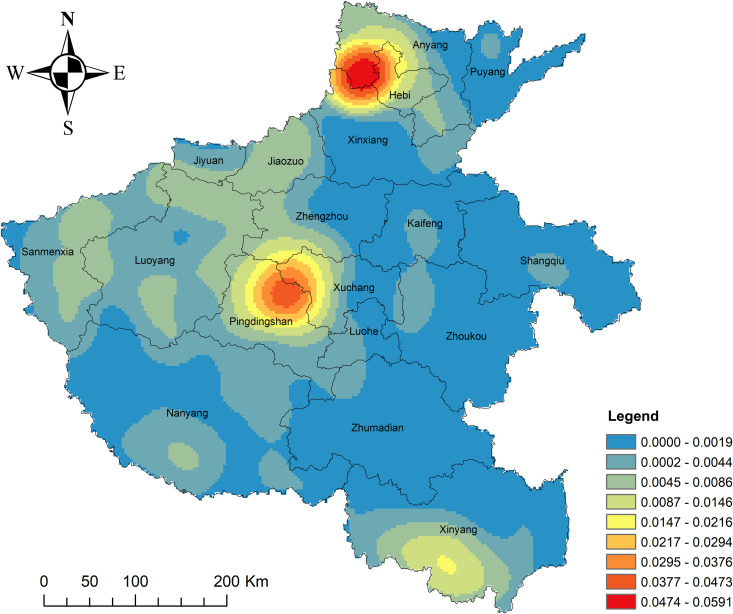
Kernel density distribution map of traditional villages (KDE bandwidth = 50 km).Source of base map: the open source map data service provided by the national basic geographic information center (http://www.ngcc.cn/ngcc/).

To optimize the analysis, we conducted a sensitivity test with bandwidths of 30, 50, 70, and 90 km (S2 Appendix, Fig 1). We selected 50 km as the optimal bandwidth because: 1) it matches Henan’s 3 core village clusters (80–150 km apart) to avoid over-segmentation/smoothing; 2) it minimized MISE (0.0021) and aligns with village density (0.0062 villages/km^2^); 3) it overlaps with historical settlement service radii (50–80 km around ancient capitals). A Gaussian kernel function with an output cell size of 3 km × 3 km was applied. The analysis was restricted to the administrative boundary of Henan, using the “Extract by Mask” function in ArcGIS to exclude areas outside the study region.

#### 2.3.7. Spatial lag model.

The Spatial Lag Model (SLM), also known as the Spatial Autoregressive Model (SAR), was applied to account for spatial dependence and spillover effects among regions in our analysis of traditional village distribution. The SLM incorporates a spatially lagged dependent variable into the standard linear regression framework, allowing us to measure how the characteristics of one region influence neighboring regions. The model is described as follows:


$$\begin{array}{c} y=\rho Wy+X\beta +\varepsilon \end{array}$$ (8)

Where *y* is the dependent variable, *W* is the spatial weight matrix defining neighborhood relationships, Wy is the spatial lag term, ρ is the spatial regression coefficient, X is the matrix of explanatory variables, β is the vector of coefficients, and ε is the random error term [16].

#### 2.3.8. Geographical detectors.

The geographical detectors methodology was applied to quantify the spatial heterogeneity of traditional village distributions across Henan Province. This approach divides the study area into multiple sub-regions and evaluates whether the variance within each sub-region is smaller than the total variance across the entire region [26]. A greater difference between these variances indicates a stronger spatial heterogeneity. The method is mathematically expressed as follows:


$$\begin{array}{c} q=\text{1}-\frac{\sum_{i=\text{1}}^{L}N_{h}\overset{\text{2}}{\underset{h}{\sigma }}}{N\sigma^{\text{2}}} \end{array}$$ (9)

Where q represents the explanatory power of a given factor on spatial heterogeneity, *L* is the number of sub-regions, $N_{h}$ and $N\sigma^{\text{2}}$ are the population and variance across the entire study area, respectively [26]. Higher q values indicate that the factor strongly explains the spatial variation of village distribution. By applying Geographical Detectors, we were able to assess how natural, historical, and socioeconomic factors contribute to the observed spatial patterns of traditional villages in Henan.

#### 2.3.9. Factor detection and interaction detection.

Factor detection quantifies the explanatory power of individual factors on the spatial distribution of traditional villages. In this framework, *h* represents the number of layers of either the dependent variable *y* or the independent variable *x*; *N* is the total number of samples, and *N*_*h*_ is the number of samples in the *h*-th layer. $\sigma^{\text{2}}$ is the variance of *y* across the entire study area, and $\overset{\text{2}}{\underset{h}{\sigma }}$ is the variance within the *h*-th layer. The *q* value ranges from 0 to 1, where higher values indicate stronger explanatory power of the independent variable *x* on the dependent variable *y*.

Interaction detection assesses how pairs of independent variables jointly affect the distribution of traditional villages. By comparing the q values of variable interactions to those of the individual variables, we can determine whether factors exhibit enhancement, weakening, or independent effects on the dependent variable. This approach reveals the complex interdependencies among natural, cultural, and socioeconomic influences on village distribution. The mathematical formulation and classification of interaction types are provided in Table 2 [26].

**Table 2:** GeoDetector and spatial lag model (SLM) associated with traditional village distribution.

	Spatial Lag Model (SLM) Regression Coefficient	Standard Error	z-value	P-value	95% CI	GeoDetector q-statistic	GeoDetector p-value
Elevation	0.000	0.000	0.769	0.442	−0.000 ~ 0.000	0.102	0.000
River	0.000	0.000	0.454	0.649	−0.000 ~ 0.000	0.0016	0.2857
GDP	0.000	0.000	0.541	0.588	−0.000 ~ 0.000	0.0370	0.0000
City	0.000	0.000	0.612	0.541	−0.000 ~ 0.000	0.0022	0.1364
Wy(Kernel)	0.900**	0.242	3.715	0.000**	0.425 ~ 1.374		
Pseudo R²			0.779				
Spatial Pseudo R²			0.045				

* p < 0.05 ** p < 0.01.

### 2.4. Influencing-factor analysis

To examine the factors associated with the spatial distribution of traditional villages, four variables were selected: elevation, distance to rivers, GDP, and distance to county-level central cities. Elevation was extracted from the DEM dataset, distance-to-river and distance-to-city variables were calculated in GIS using Euclidean distance, and GDP data were obtained from official statistical sources and matched to the corresponding spatial units. These continuous variables were grouped into classes following the intervals shown in Figs 5–8. Factor effects were evaluated inferentially using GeoDetector and the spatial lag model (SLM). Variables that were not statistically significant in the SLM were interpreted cautiously and were not treated as confirmed independent determinants.

Guided by the above conceptual framework, the selected variables represent three dimensions of village persistence: environmental constraint (elevation and river proximity), uneven urbanization and development pressure (GDP and distance to county-level central cities), and historically accumulated spatial structure, captured through temporal reconstruction and spatial autocorrelation.

## 3. Spatial and temporal distribution characteristics

### 3.1. Spatial distribution characteristics

#### 3.1.1. Spatial distribution type.

We analyzed the spatial distribution of traditional villages in Henan Province to evaluate their connectivity and clustering characteristics. Using ArcGIS 10.2, we calculated that the observed average neighbor distance among villages is approximately 9,967 meters, compared to the expected distance of 16,552 meters under a random distribution (*D* = 0.006205 villages/km^2^, r_E_ = 16, 550m). The resulting nearest neighbor index (R = 0.6017) is less than 1, indicating a significant clustering tendency. A further statistical test yielded a Z-value of −12.86, well below the critical threshold of −2.58, with a P-value of 0, confirming that the clustering pattern was highly statistically significant. These results demonstrate that traditional villages in Henan are not randomly distributed but rather exhibit pronounced agglomeration.

To complement the nearest neighbor analysis and address ongoing debates regarding its applicability for point patterns, we employed the coefficient of variation (CV) of Thiessen polygon areas as a supplementary measure. The Thiessen polygons generated in ArcGIS 10.2 yield a CV of 170.49%, substantially exceeding the 64% threshold commonly used to indicate clustering. This finding corroborates the results from the nearest neighbor index, confirming a clear clustered spatial distribution of traditional villages across Henan Province. By integrating multiple spatial metrics, our analysis provides a robust and comprehensive understanding of village aggregation patterns.

#### 3.1.2. Spatial distribution pattern.

ArcGIS kernel density estimation was applied to analyze the spatial distribution patterns of 1,035 traditional villages in Henan Province (Fig 1). And the analysis identified three distinct regional hotspots: a primary high-density core area near Anyang and Hebi in northern Henan; a secondary cluster in the eastern part of Pingdingshan in central Henan; and a concentrated cluster around Xinyang in southern Henan. In contrast, western Henan exhibits a relatively uniform and dispersed distribution of villages, whereas eastern Henan shows a sparse village presence, reflecting an overall uneven spatial pattern. The northern and central areas host the highest concentrations of traditional villages, followed by southern Henan, with western Henan demonstrating a more balanced distribution.

Several natural and socioeconomic factors contribute to this pattern. The prevalence of mountains and hills across central, southern, and northern Henan, combined with limited historical transportation infrastructure, has minimized external disruptions, preserving many tranquil and intact traditional villages. Western Henan, located within the transitional zone from the second to third topographic steps of China, features complex and varied landforms with significant elevation differences, supporting a more even village distribution. Conversely, eastern Henan, characterized by flat terrain and an extensive transport network, has historically experienced rapid penetration of external influences, posing challenges to the preservation of traditional villages. Additionally, the frequent historical flooding of the Yellow River in eastern Henan further complicates conservation efforts, reducing the likelihood of maintaining villages in their original state over time.

#### 3.1.3 Geographical distribution.

**Fig 2:**
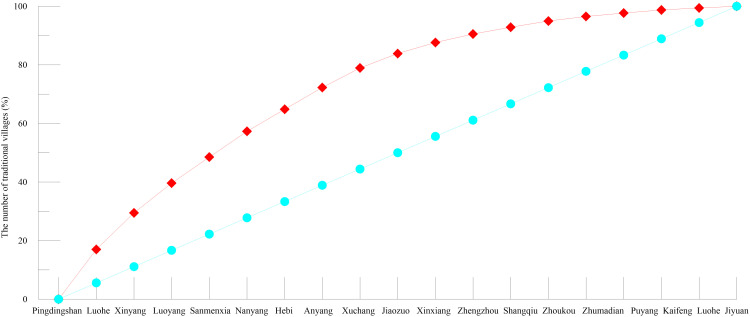
Lorenz Curve of the spatial distribution of traditional villages in Henan Province.

The Henan Province contains 1,035 nationally recognized traditional villages distributed across 158 counties and districts, revealing a markedly uneven geographical pattern. We calculated a geographical concentration index (G) of 20.09 using formula (5), which substantially exceeds the theoretical average of 7.48, indicating strong spatial concentration. Consistently, the imbalance index (S), which was computed using formula (6), reached 0.74, which is far above 0, confirming a high degree of spatial inequality in village distribution. The Lorenz graph (Fig 2) further visualizes this imbalance, showing that traditional villages are heavily clustered in a limited number of cities. Specifically, Pingdingshan, Xinyang, Luoyang, Sanmenxia, Nanyang, Hebi, and Anyang together account for approximately 70% of all traditional villages in the province, underscoring the dominance of these areas in preserving traditional settlements. This concentration is attributed to historical (imperial capitals’ radiation), topographic (moderate elevations and water proximity), and policy (prioritized protection) drivers, as elaborated in subsequent sections.

### 3.2 Spatial and temporal evolution characteristics

The spatial and temporal evolution of traditional villages highlights the long-standing role of Henan Province as a core region of ancient Chinese civilization. Archeological evidence from the pre-Qin period, including that associated with the Pei Ligang, Yangshao, and Longshan cultures, demonstrates early and continuous human settlement in the region. During the Xia, Shang, and Zhou Dynasties, Henan functioned as a political, economic, and cultural center, fostering sustained advances in social organization, technology, and material culture that shaped early village formation. This developmental momentum continued into the later dynasties. Although the Tang capital was located in neighboring Shaanxi and the Song capital in Kaifeng, Henan remained deeply integrated into national political and economic systems, supporting the emergence of influential traditional settlements, such as Longmen Village in Luoyang and Zhuxian Town in Kaifeng. During the Ming and Qing Dynasties, Henan’s designation as a major grain-producing region further stimulated the expansion and consolidation of rural settlements, including Chaohua Village in Zhengzhou and Matoujian Village in Anyang. During the Anti-Japanese War, the strategic location of Henan reinforced its role as a political and transportation hub, with western Henan designated as one of China’s 19 anti-Japanese base areas, adding a modern historical layer to village evolution.

Building on the unique geographical location, economic status, and historical depth of Henan, this study uses traditional villages as focal points to examine regional spatial-temporal dynamics. We divided village development into four key stages: the pre-Qin formative era, the flourishing Tang, Song, and Yuan dynasties, the prosperous Ming and Qing dynasties, and the post-Qing period, which was shaped by the War of Resistance against Japan. Each stage reveals distinct features of the spatial-temporal evolution of villages. Given the long and complex histories of many villages, we prioritized comprehensive chronological reconstruction and successfully compiled formation period data for all 1,035 traditional villages included in the study. Specifically, 270 villages were from the pre-Qin period, 79 from the Tang, Song, and Yuan dynasties, 561 from the Ming and Qing dynasties, and 125 from the post-Qing dynasty. These data constitute a crucial foundation for our research.

**Fig 3:**
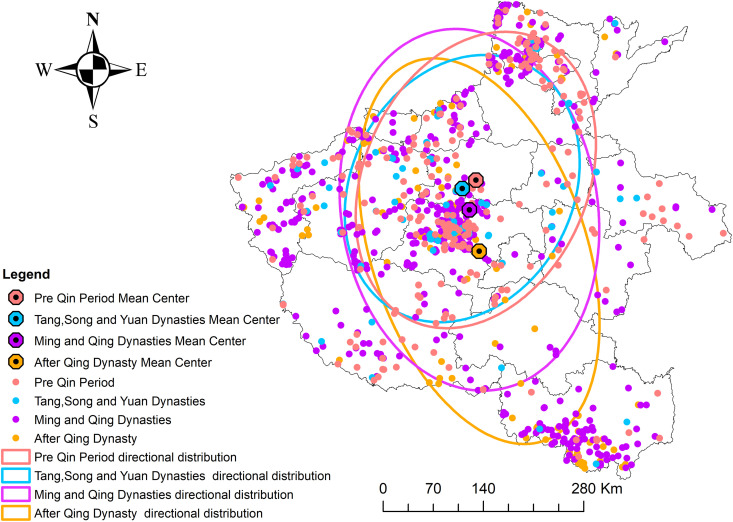
Overall evolution of the spatial pattern of traditional villages in Henan Province. Source of base map: the open source map data service provided by the national basic geographic information center (http://www.ngcc.cn/ngcc/).

In Fig 3, the standard deviation ellipses correspond to four chronological stages: the pre-Qin period, the Tang-Song-Yuan period, the Ming and Qing period, and the post-Qing period. The ellipses in different colors represent different stages, reveals clear shifts in the directional structure and spatial extent of traditional villages across historical periods. During the pre-Qin era and the Tang-Song-Yuan dynasties, village distributions followed a dominant southwest-northwest orientation, reflecting the early influence of Yin Shang, Weiguo, and Tang cultural cores on agrarian development and rural settlement formation in areas such as Anyang, Hebi, and Pingdingshan. In the pre-Qin period, the ellipse covered a slightly larger area but exhibited a lower length-to-width ratio, indicating relatively sparse yet geographically dispersed settlements. In contrast, villages in the Tang-Song-Yuan period displayed stronger directionality, accompanied by a pronounced southwestward shift in the center of gravity. A marked reorientation occurred during the Ming and Qing dynasties when the ellipse aligned along a northwest-southeast trajectory. This transition coincided with important agricultural and hydrological changes in southern Henan, particularly in Xinyang, including the expansion of paddy cultivation, crop restructuring, and the development of local water conservancy works along the Huaihe and Ruhe river systems, which likely enhanced agricultural productivity and supported settlement growth [27,28]. Entering the Qing period, the ellipse contracted spatially while the major-to-minor axis ratio increased, suggesting stronger directional clustering despite broader regional coverage. In the post-Qing era, village distributions exhibited even stronger directionality, with the geometric center shifting further southeast, reflecting continued population redistribution and evolving human-land interactions.

**Fig 4:**
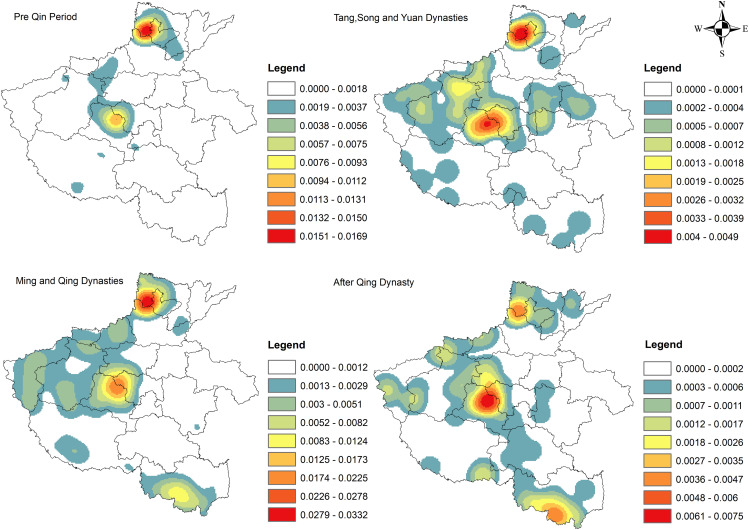
Kernel density distribution map of traditional villages for the different periods (KDE bandwidth = 50 km).Source of base map: the open source map data service provided by the national basic geographic information center (http://www.ngcc.cn/ngcc/).

Kernel density analysis (Fig 4) indicates pronounced temporal variation in the spatial distribution of traditional villages across successive historical periods in Henan Province. During the pre-Qin period, villages clustered most strongly in northern Henan, with Anyang and Hebi forming the dominant cores. In the Tang, Song, and Yuan dynasties, high-density areas expanded into northern and central Henan, with central Henan emerging as the primary concentration zone. This shift reflects the region’s sustained role as a political, economic, and cultural center, reinforced by the capital functions of Luoyang and Kaifeng, which promoted population concentration and settlement expansion [1,11,16]. In the Ming and Qing dynasties, village development was distributed across three regions: central, northern, and eastern Henan, and northern Henan gradually assumed a leading role. The decline of Kaifeng and Luoyang following the fall of the Northern Song Dynasty weakened central Henan’s national political status, reducing its relative socioeconomic advantage and village density. Simultaneously, repeated warfare and the strategic vulnerability of the central plains likely contributed to population redistribution toward relatively sheltered areas, including parts of northern Henan, thereby reinforcing northern Henan as an important settlement core [11,18].

During the Ming Dynasty, agricultural transformation further reshaped village distribution, particularly in southeastern Henan (Xinyang), where rice cultivation, cotton planting, and large-scale water conservancy projects along the Huaihe and Ruhe rivers substantially increased agricultural productivity and population. These changes marked the emergence of southeastern Henan as an important new development zone for traditional villages. In the post-Qing period, the kernel density results again identified three major concentration centers in central, northern, and eastern Henan, with central Henan reasserting itself as the dominant hub. This resurgence is likely associated with the administrative and economic prominence of Kaifeng and Zhengzhou, favorable policy support, and relatively strong agricultural resources, which together helped sustain traditional village continuity in central Henan [1,8,9,16].

## 4. Influencing factors

### 4.1. Topographic conditions associated with the distribution of traditional villages

**Fig 5:**
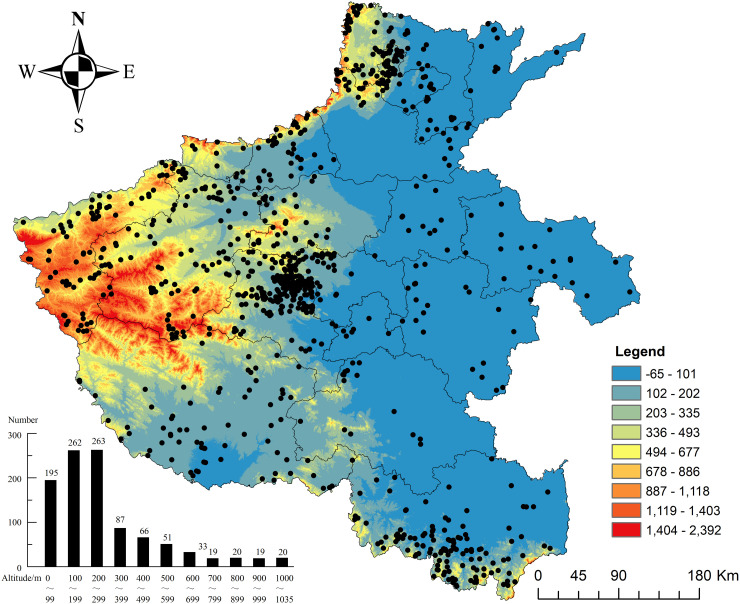
Distribution of traditional villages at various elevations in Henan Province. Source of base map: the open source map data service provided by the national basic geographic information center (http://www.ngcc.cn/ngcc/). Digital Elevation Model (DEM) digital elevation data were obtained from the Geospatial Data Cloud platform of the Chinese Academy of Sciences (https://www.gscloud.cn).

Human settlement patterns closely reflect the constraints and opportunities imposed by the natural environment. Situated at the confluence of China’s second and third topographical regions, Henan Province exhibits pronounced geomorphological characteristics, including extensive plains, low mountains, basins, and hills. These features are closely linked to the spatial distribution and preservation of traditional villages. Analysis of village distribution across various altitudes (Fig 5) shows a strong concentration within the 100–300 meters range, which contains 720 traditional villages, accounting for 69.6% of the total sample (n = 1,035). Village numbers increase steadily with altitude up to 300 meters, peaking at 200–300 meters with 203 villages, before declining sharply at higher elevations. Beyond 300 meters, the number decreases sharply, with only 87 villages (8.4%) between 300 and 400 meters and just 20 villages (1.9%) above 1,000 meters. These results indicate a clear nonlinear relationship between altitude and village distribution rather than a simple monotonic trend.

Extreme altitudes, both high and low, appear unfavorable for sustaining traditional villages. In the mountainous areas of western Henan (light blue/low-density zones in Fig 5), rugged terrain, steep elevation variations, limited arable land, and relatively harsh living conditions constrain agricultural production and inhibit the development of large-scale village settlements. Conversely, the low-lying plains of eastern Henan, including Puyang, Shangqiu, Kaifeng, and Zhoukou (dark blue/low-density clusters in Fig 5), benefit from flat terrain and developed transportation networks but remain highly vulnerable to external disturbances.

Overall, topographic conditions show a clear descriptive association with the distribution of traditional villages in Henan Province, although their independent effect should be interpreted cautiously in light of the spatial regression results. Elevations between 100 and 300 meters offer an optimal balance among agricultural viability, flood risk mitigation, and defensive requirements, thereby supporting sustained settlement over long historical periods. Section 4.5 further examines the statistical significance of this relationship, as well as its interaction with other driving factors and spatial autocorrelation effects.

### 4.2. Hydrological conditions associated with the distribution of traditional villages

**Fig 6:**
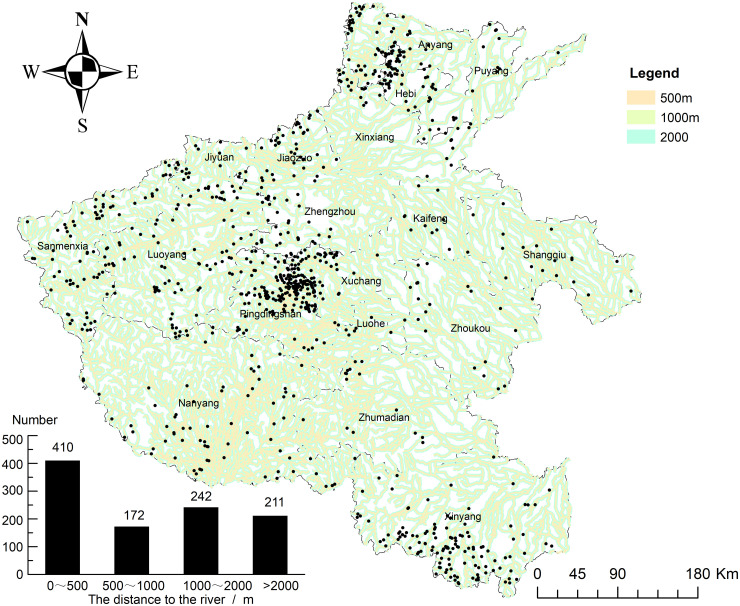
Association between the distribution of traditional villages and rivers. Source of base map: the open source map data service provided by the national basic geographic information center (http://www.ngcc.cn/ngcc/). River data from the 1:250,000 national basic geographic database provided by the national geographic information resources catalogue service system (www.webmap.cn).

Water systems form a fundamental environmental framework that shapes human habitation, agricultural production, and long-term population stability. Historically, communities have preferentially established villages near rivers, streams, and other surface water bodies to secure domestic water supply, irrigation, and transportation access. To examine the influence of hydrological conditions on the spatial distribution of traditional villages in Henan Province, this study employed ArcGIS 10.2 to calculate the distance between each village and its nearest water system. Fig 6 presents the spatial pattern of the villages relative to the water networks and the corresponding distance-based frequency distribution. The results demonstrate a clear spatial dependence of village locations on proximity to water resources.

As shown in the histogram inset of Fig 6, 410 villages (39.61%) lie within 0–500 meters of a water system, while an additional 172 villages (16.62%) fall within the 500–1000 meter range. More than half of all traditional villages are located within 1000 meters of water sources, underscoring the strong hydrological dependence of settlement distribution. Beyond this threshold, 242 villages (23.38%) occur at distances of 1000–2000 meters, and 211 villages (20.39%) are situated more than 2000 meters from water sources. When combined with the spatial distribution in Fig 6, high-density clusters, particularly around Pingdingshan in central Henan, were clearly concentrated within 1000 meters of the water system and coincided with moderate-altitude regions (500–1000 meters). This spatial convergence indicates a synergistic interaction between hydrological accessibility and favorable topographic conditions in shaping village persistence.

From a multifaceted perspective, the spatial clustering of traditional villages near water sources reflects the adaptive strategies of traditional societies. In terms of basic survival, villages developed in the absence of modern water supply systems and therefore required reliable access to water sources or basic facilities such as wells and ditches within a 2000-meter radius to fulfill the water needs of both humans and livestock. In Henan, 824 villages (79.61% of the total) lie within 2000 meters of a water source, indicating that hydrological proximity is a fundamental condition for sustaining human and livestock populations. From a production perspective, Henan’s long-standing dependence on agriculture further reinforced this pattern. Villages located within 0–500 meters of water sources (410 villages) commonly occupy valley terraces, particularly in central Henan (Fig 6), where fertile alluvial soils and convenient irrigation infrastructure support stable agricultural production and long-term settlement continuity. Hydrological proximity also reflects safety and cultural practices. Villages situated 500–1000 meter from water bodies (172 villages), including those in Zhoukou and Shangqiu in eastern Henan, appear to balance water accessibility with flood-risk avoidance, particularly in relation to the Yellow River and Huaihe rivers. This intermediate distance reduces exposure to catastrophic flooding while preserving functional access to water resources. Culturally, Henan’s role as a core region of early Chinese civilization fostered a persistent tradition of “living by water,” where rivers functioned as corridors for trade, communication, and cultural exchange, especially in western Henan. These hydrologically oriented settlement patterns do not operate in isolation, but they interact with topographic, historical, and socioeconomic factors. Their statistical significance and interaction mechanisms are examined in detail in Section 4.5. In conclusion, hydrological conditions are significantly correlated with the spatial distribution of traditional villages in Henan Province. This correlation is embedded in the synergistic adaptation of these villages to both water resources and topographic environments, and further statistical validation is provided in Section 4.5.

### 4.3 Socio-economic conditions associated with the distribution of traditional villages

**Fig 7:**
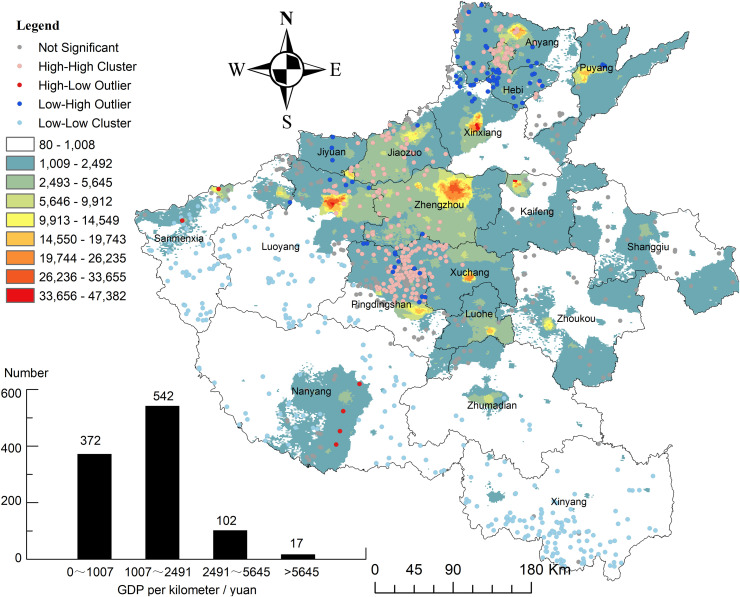
Association between the distribution of traditional villages and regional economic development. Source of base map: the open source map data service provided by the national basic geographic information center (http://www.ngcc.cn/ngcc/). GDP were obtained from the Resource and Environment Science and Data Center of the Chinese Academy of Sciences (www.resdc.cn).

Socio-economic conditions play a critical role in shaping the spatial distribution and long-term preservation of traditional villages by mediating pressure from development and land-use transformation. To evaluate this relationship, this study employed 1 km^2^ GDP grid data obtained from the Geographic Detection Cloud Platform for the years 1995, 2000, 2005, 2010, and 2015, aggregated into 5-year averages to reduce short-term economic volatility. Fig 7 presents the resulting spatial patterns, including the LISA cluster maps and the frequency distribution of villages across the GDP intervals.

As shown in the histogram inset of Fig 7, 372 villages (35.95%) occur in areas with GDP values between 0 and 1,007 yuan/km^2^ while an additional 542 villages (52.36%) lie within the 1,007−2,491 yuan/km^2^ range. In contrast, only 102 villages (9.86%) are located in moderately developed areas (2,491 and 5,645 yuan/km^2^), and just 17 villages (1.64%) appear in regions exceeding 5,645 yuan/km^2^. Overall, 914 villages, representing 88.3% of the total sample, are located in areas with GDP levels below 2,500 yuan/km^2^. This pattern corresponds closely with the LISA spatial clustering results: Low-Low Clusters (low GDP and low village density) dominate eastern Henan, particularly in Zhoukou and Shangqiu, while prominent village concentrations in central Henan, such as around Pingdingshan, exhibit Low-High patterns (low GDP and high village density). These findings indicate that traditional villages are more likely to persist in economically underdeveloped regions, where slower urban expansion and reduced industrial encroachment limit the erosion of historical settlement forms.

This distribution pattern reflects the adaptive characteristics that underlie the preservation of traditional villages. In moderately underdeveloped regions, with GDP levels ranging from 1,007–2,491 yuan/km^2^ and encompassing 542 villages, the relatively slow pace of rural urbanization reduces external disturbances, such as large-scale construction, land conversion, and cultural homogenization. These conditions foster a comparatively stable socio-spatial environment that supports the preservation of ancient architecture, traditional customs, and indigenous lifestyles. As shown in Fig 7, these areas, represented by clustered black points in central Henan, represent the core habitats of traditional villages. Conversely, zones characterized by extremely low GDP levels (0–1,007 yuan/km^2^), including 372 villages, face a different set of constraints. In areas such as western Nanyang (Fig 7), harsh living conditions, limited infrastructure, and sustained population outflow undermine the long-term viability of traditional settlements. The dispersed distribution of villages, indicated by scattered black points, suggests that extreme poverty undermines the sustainability of traditional settlements. In high GDP regions (>5,645 yuan/km^2^), comprising only 17 villages, rapid urbanization and modern development have led to the displacement of traditional settlements. The few remaining villages, identified as High-Low outliers and marked by red points in Fig 7, persist primarily because of specific cultural policies. These patterns indicate that the concentration of traditional villages in low-GDP areas is not a random occurrence but reflects a structured response to varying development pressures. The statistical significance of this relationship, along with its interactions with other influencing factors, is further examined in Section 4.5.

In conclusion, the spatial distribution of traditional villages in Henan Province shows a descriptive association with regional socio-economic development; however, this relationship was not statistically significant as an independent effect in the SLM and should therefore be interpreted cautiously. Most villages are concentrated in moderately underdeveloped areas (1,007 ~ 2,491 yuan/km^2^ where slower urbanization processes facilitate their preservation. Additional statistical validation of these synergistic mechanisms is provided in Section 4.5.

### 4.4 Distance to county-level central cities associated with the distribution of traditional villages

**Fig 8:**
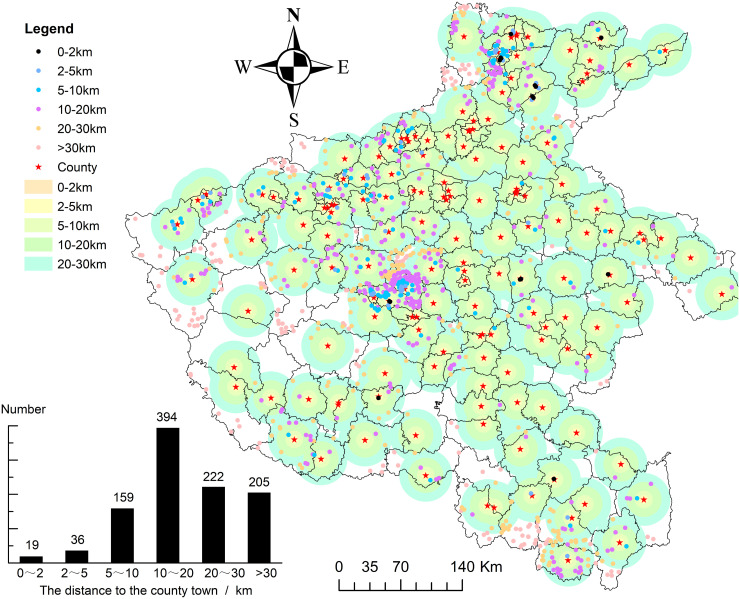
Relationship between the distribution of traditional villages and regional central cities. Source of base map: the open source map data service provided by the national basic geographic information center (http://www.ngcc.cn/ngcc/).

To examine the relationship between the traditional village distribution and regional central cities, this study selected 108 county towns in Henan Province as reference points. Using ArcGIS, the Euclidean (straight-line) distance from each traditional village to its nearest county town was calculated. The resulting spatial patterns and village frequencies across distance intervals are presented in Fig 8, which combines color-coded distance zones with histogram statistics.

As shown in the histogram inset of Fig 8, only 19 traditional villages (1.84% of the total 1,035 villages) are within 0–2 km of county towns, while 36 villages (3.48%) fall within the 2–5 km range. A larger number of villages, 159 (15.37%), are situated 5–10 km away. The highest concentration occurs within the 10–20 km interval, which contains 394 villages (38.07%). Beyond this range, 222 villages (21.45%) are located 20–30 km away, and 205 villages (19.71%) are located more than 30 km from county towns. Fig 8 shows that high-density village clusters in northern and central Henan largely coincide with the 10–20 km distance zone, whereas areas immediately surrounding county towns (0–2 km), represented by light orange zones, exhibit an extremely sparse distribution of villages. This pattern indicates a clear distance-dependent relationship between traditional village distribution and county-level urban centers.

The distribution of traditional villages in Henan Province reflects a dynamic balance between urban influence and the preservation of historical settlements. Within 0–5 km of county towns, only 55 villages (5.32%) result from rapid urban expansion. In these areas, intensive modern construction, demolition, and cultural homogenization have displaced or transformed traditional settlements, producing the blank/light orange zones surrounding county towns in Fig 8. The 10–20 km range hosts 394 villages, forming the core distribution zone. Villages within this interval benefit from convenient access to essential urban services, such as markets, schools, and public facilities, while maintaining sufficient distance to avoid direct interference from urban development. This combination of accessibility and autonomy creates a stable environment that supports the preservation of traditional architecture, settlement layouts, and cultural practices, as illustrated by the dense, multi-colored clusters in central Henan in Fig 8. Beyond 30 km from county towns, although 205 villages experience minimal urban disruption, they face logistical and resource-related challenges, including limited transportation infrastructure and access to services. These constraints contribute to a more scattered and less dense settlement pattern, exemplified by the sparse points along the southern edges of Henan in Fig 8.

Collectively, these observations indicate a strong distance-dependent effect on village distribution, in which the 10–20 km buffer emerges as the optimal zone for sustaining traditional settlements. The underlying statistical relationships and interactive mechanisms shaping this pattern are rigorously analyzed in Section 4.5, reinforcing the significance of proximity to county-level central cities in mediating the balance between preservation and urban influence.

### 4.5 Inferential verification and integrated analysis of influencing factors

To complement the descriptive patterns presented in Sections 4.1–4.4, we applied GeoDetector and the spatial lag model (SLM) for inferential analysis. The grouped distributions in Figs 5–8 were used only to illustrate descriptive tendencies, whereas the statistical significance and explanatory strength of factors were evaluated in this section. The GeoDetector quantified the explanatory power of individual factors, such as topography, hydrology, socioeconomic conditions, and distance to central cities at the county-level, and assessed the interactions between these factors. SLM accounted for spatial autocorrelation, capturing spillover effects across neighboring regions. Integrating these approaches enabled us to create a cohesive framework linking observed distribution patterns to underlying causal processes, confirming the significance of key factors and revealing synergistic influences that shape settlement patterns.

#### 4.5.1. Relative importance of single factors.

**Fig 9:**
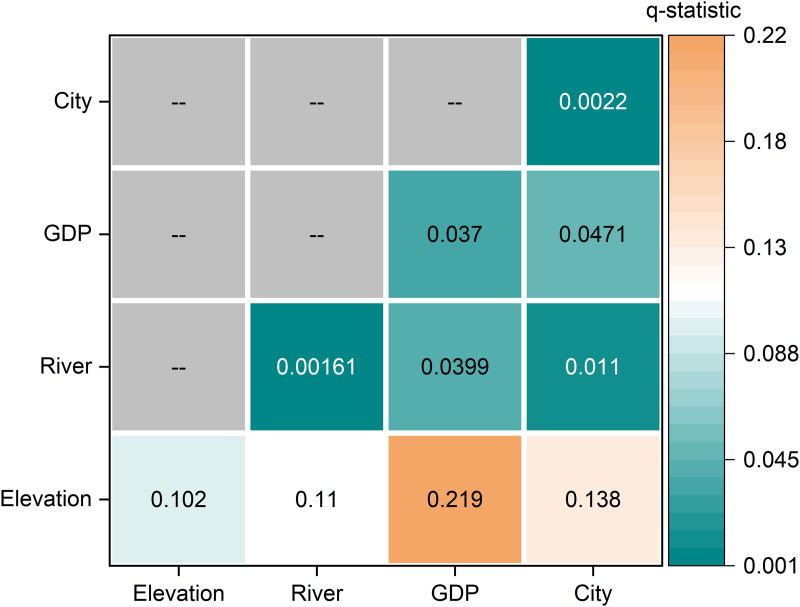
GeoDetector-based factor detection and interaction effect analysis of variables associated with traditional village distribution.

Factor detection quantified the explanatory power of each variable for the spatial heterogeneity of traditional villages in Henan Province (Fig 9). In the GeoDetector results, elevation showed the highest q value (q = 0.102, P < 0.001), whereas GDP showed a smaller but statistically significant q value (q = 0.037, P < 0.001). These results suggest that elevation and GDP are associated with the observed spatial heterogeneity at the descriptive stratification level. However, after spatial autocorrelation was controlled for in the SLM, neither elevation (P = 0.442) nor GDP (P = 0.588) remained statistically significant. Therefore, their effects should not be interpreted as robust independent determinants, but rather as contextual factors whose influence may depend on spatial dependence and interactions with other variables.

Hydrological conditions, represented by river distance, showed a very low q value (q = 0.0016, P = 0.2857), and distance to county-level central cities also exhibited weak explanatory power (q = 0.0022, P = 0.1364). These results indicate that the independent effects of these variables are limited when considered separately. Taken together, the factor detection and SLM results suggest that the distribution of traditional villages is better understood as the outcome of combined influences and spatial spillover rather than isolated single-factor effects.

#### 4.5.2. Synergistic interaction effects of multiple factors.

The interaction analysis revealed that all examined factor pairs produced enhancement effects, demonstrating that combined environmental and socioeconomic conditions better explain the spatial distribution of traditional villages than individual factors alone. These results support the adaptive patterns described in Sections 4.1–4.4.

For example, the Elevation×GDP interaction exhibited non-linear enhancement, with a q-statistic of 0.219 (P < 0.001). The combined explanatory power of 21.9% significantly exceeds the sum of the independent effects of elevation (10.2%) and GDP (3.7%), highlighting a synergistic influence. This interaction corresponds directly to the core distribution pattern, where 69.6% of villages occupy elevations of 100–300 meters and 52.36% lie in areas with a GDP of 1,007–2,491 yuan/km^2^. This “moderate terrain + low urbanization” niche effectively balances agricultural productivity with minimal external disturbances, thereby establishing a critical zone for the preservation of traditional architecture and local culture.

For the Elevation×River distance interaction, bilinear enhancement was observed (q-statistic = 0.11, P < 0.01), confirming the synergistic adaptation patterns observed in Sections 4.1 and 4.2. High-density clusters, particularly in central Henan, predominantly occur in areas characterized by moderate altitudes (500–1000 m) combined with proximity to water sources (0–1000 m). The terrain reduces flood risk, while nearby rivers and streams sustain agricultural production, explaining why 79.61% of villages lie within 2,000 meters of water.

Similarly, the GDP x Distance to County-Level Central Cities Interaction exhibited bilinear enhancement (q-statistic = 0.0471, P < 0.05), reinforcing the patterns identified in Sections 4.3 and 4.4. Villages within the 10–20 km range of county towns, which account for 38.07% of the total, predominantly coincide with areas of low GDP (<2500 yuan/km^2^). Moderate accessibility to urban centers provides essential services, whereas limited economic development minimizes demolition and cultural homogenization, creating a stable environment conducive to the preservation of traditional villages.

#### 4.5.3. Spatial autocorrelation correction and verification.

The Global Moran’s I test (I = 0.130964, Z = 8.578887, P = 0) revealed a significant positive spatial autocorrelation, validating the application of the SLM to account for spatial dependence. The spatial lag term (Wy(Kernel)) was highly significant (β = 0.900, P < 0.01), indicating a pronounced spatial spillover effect, whereby high-density village clusters in northern and central Henan facilitate the emergence of secondary clusters in adjacent regions through cultural diffusion, population migration, and shared agricultural practices. After controlling for spatial autocorrelation, regression coefficients for individual factors—elevation (β = 0.000, P = 0.442), river distance (β = 0.000, P = 0.649), GDP (β = 0.000, P = 0.588), and urban distance (β = 0.000, P = 0.541)—were all statistically insignificant. These results confirm that the observed spatial patterns, such as the concentration at villages at elevations of 100–300 meters and the 10–20 kilometers optimal range from county towns, arise not from isolated determinants but from synergistic interactions and spatial spillover effects.

## 5. Discussion

This study analyzed 1,035 traditional villages in Henan Province, a pivotal region in China’s Central Plains civilization, to examine their spatiotemporal distribution and underlying driving mechanisms. By applying a combination of spatial and statistical analyses, the study elucidates how natural conditions, socioeconomic factors, and historical legacies jointly shape village distribution. These findings address a notable gap in systematic studies of the Central Plains, which has received less attention than Southwest China, the Tibetan Plateau, and coastal regions.

### 5.1. Spatial distribution pattern: Between natural adaptation and cultural continuity

The spatial clustering of traditional villages in northern, central, and southern Henan reflects deliberate adaptation to environmental and socio-cultural constraints rather than random settlement. More broadly, this pattern can be understood through a settlement persistence perspective: traditional villages are not simply remnants of the past, but spatial products of long-term adaptation in which environmental suitability and cultural continuity reinforce each other. Villages consistently occupy areas that maximize agricultural productivity while minimizing ecological risks, such as flooding in eastern plains and extreme conditions in mountainous western regions. Northern Henan’s proximity to ancient capitals, such as Anyang, has preserved and transmitted Yin-Shang cultural heritage, whereas central Henan’s historical role as a political and economic hub has maintained settlement continuity through successive dynasties. This spatial configuration underscores a long-standing integration of natural adaptation and cultural continuity, where human activity, environmental suitability, and heritage preservation collectively shape the observed patterns.

In eastern Henan, the sparse distribution of traditional villages reflects the combined influence of natural hazards and human activities. Recurrent flooding of the Yellow River repeatedly destabilized settlements, limiting long-term village persistence. Rapid urbanization and intensive agricultural development have accelerated the homogenization of rural landscapes, further constraining village continuity. This scenario contrasts with the high-altitude regions of Southwest China, where topographic barriers primarily drive low settlement density, highlighting the unique human-land relationship in the Central Plains. Conversely, western Henan exhibits a more uniform and dispersed settlement pattern, as its position within the transitional terrain zone, with diverse landforms and moderate altitudes, supports stable, adaptable village networks that align with complex environmental conditions. These patterns collectively illustrate how traditional villages in Henan have evolved under synergistic environmental and socioeconomic pressures.

### 5.2. Spatiotemporal evolution: Responding to historical dynamics and agricultural transformation

The shift in traditional village distribution from a southwest-northeast to a northwest-southeast orientation represents the adaptive responses of rural settlements to significant historical and agricultural transformations in the Central Plains. This directional shift also reflects a path-dependent settlement process in which earlier political cores, agricultural infrastructures, and migration routes continued to shape later village survival and redistribution. Before the Ming Dynasty, settlement patterns were closely aligned with the expansion of agrarian civilization and the concentration of political resources, with ancient cultures such as the Yin-Shang and Weiguo, along with dynastic capitals like Luoyang and Kaifeng, shaping the primary settlement axis. During the Ming and Qing Dynasties, this spatial configuration shifted in response to agricultural technology and population migration. The expansion of rice and cotton cultivation, together with the development of water conservancy works in southeastern Henan, likely enhanced land productivity and attracted population growth and settlement expansion [27,28]. While central Henan experienced population outflows due to diminishing political significance and recurrent conflicts, redirecting settlements toward northern mountainous regions.

This pattern contrasts sharply with settlement evolution in Southwest China, where ethnic migration and cultural preservation exert the strongest influence, and with coastal villages, where maritime trade predominates. In the Central Plains, agricultural development and political changes have historically remained the primary drivers of village formation and spatial evolution. These findings extend the theoretical framework of traditional village spatiotemporal dynamics by highlighting the region-specific interactions between human activity, political structures, and agricultural transformation.

In addition to the environmental and socio-economic variables examined here, Henan’s historical-political context likely also shaped the long-term evolution of traditional villages. As a core region of multiple dynasties, with political centers such as Luoyang, Kaifeng, Anyang, and Zhengzhou, the province experienced repeated cycles of administrative restructuring, population concentration, and infrastructure development, which may have influenced settlement continuity and relocation [1,11,16]. However, because comparable political indicators at the village scale are difficult to reconstruct consistently across long historical periods, political factors were not included in the quantitative model and should be explored further in future research.

### 5.3. Influencing factors: Synergistic effects and spatial spillover

The analysis reveals that the spatial distribution of traditional villages in Henan Province is influenced by the synergistic interaction of multiple factors rather than a single determinant. Descriptive analyses indicate that moderate topography, hydrological accessibility, and relatively low development pressure are associated with the concentration of traditional villages. However, the SLM results suggest that these variables do not operate as strong independent linear determinants after spatial dependence is taken into account; instead, their influence is more likely expressed through combined effects and spatial spillover. This multi-dimensional interaction explains why individual variables, such as river proximity or distance to urban areas, exhibit limited explanatory power when considered in isolation but reveal substantial influence in combination. The results also demonstrate the uneven impacts of urbanization on rural cultural heritage: traditional villages are threatened not only by intensive urban expansion, but also by severe marginalization, where out-migration and weak infrastructure can erode long-term continuity.

The “moderate terrain + low urbanization” niche can be regarded as the main descriptive setting in which traditional villages are concentrated, rather than as a statistically confirmed independent preservation mechanism. This pattern highlights a critical tension in heritage conservation: rapid economic development can promote modernization while simultaneously threatening traditional spaces, whereas extreme poverty can undermine the sustainability of conservation efforts. Additionally, the optimal distance of 10–20 km from county-level central cities further illustrates this equilibrium, allowing villages to access essential services without succumbing to direct urban expansion pressures. These findings provide a practical framework for harmonizing rural revitalization with heritage preservation, emphasizing the importance of context-specific planning.

The spatial lag model reveals a pronounced spatial spillover effect, indicating that the agglomeration of traditional villages is influenced not only by local environmental adaptation but also by cultural diffusion and population migration across neighboring regions. Core clusters, such as Anyang-Hebi and Pingdingshan, have extended their influence to surrounding areas, generating contiguous cultural landscapes that reflect historical settlement dynamics. These findings emphasize the need to shift from isolated, site-specific preservation to strategies that prioritize cluster-based and contiguous-area protection in contemporary cultural heritage policies, thereby safeguarding both the physical settlements and their broader socio-cultural context.

At the village level, the relative importance of topography and hydrology varies across regions. Villages in mountainous and hilly zones are more strongly constrained by elevation and terrain, villages in river-adjacent plains are more closely associated with hydrological accessibility, and many villages in central and southern Henan reflect the combined influence of both factors. This also indicates that no single environmental factor dominates all traditional villages uniformly across Henan.

### 5.4. Practical implications for protection and sustainable development

The findings of this study provide actionable guidance for the preservation and sustainable development of traditional villages in Henan Province. In the three identified high-density hotspots, protection strategies should prioritize the integration of settlements with their surrounding natural environments. In northern Henan, interventions should focus on reinforcing connections between villages and the legacy of ancient capital culture to strengthen local cultural identity. In central Henan, policies must carefully balance agricultural development with architectural preservation, preventing excessive commercialization while maintaining rural vitality. Southern Henan, with its rich ecological resources, presents opportunities to develop eco-cultural tourism that harmonizes heritage preservation with sustainable local economic development.

In moderately underdeveloped regions, slowing homogenized urbanization is critical. Policymakers should promote “culture-led rural revitalization,” prioritizing traditional crafts, intangible heritage safeguarding, and adaptive reuse of historic buildings [2,8]. In Eastern Henan, where traditional villages remain sparse, initiatives should integrate flood control heritage with ecological restoration to protect the remaining villages and, where appropriate, reconstruct culturally resonant landscapes. The demonstrated spatial spillover effect indicates the potential effectiveness of cluster-based protection approaches, such as establishing contiguous traditional village protection zones rather than relying solely on isolated site preservation [8,14]. Beyond Henan, these findings suggest that in historically layered and rapidly urbanizing regions, traditional village conservation is most effective when it is organized around settlement clusters, aligned with environmental suitability, and targeted at areas where development pressure is present but not yet overwhelming. This implication may be relevant to other inland agricultural regions undergoing similar rural transformation.

## 6. Conclusions

This study applied ArcGIS spatial analysis, GeoDetector, and the SLM to systematically investigate the spatiotemporal distribution and underlying mechanisms of 1,035 officially recognized traditional villages in Henan Province, a central area of China’s Central Plains civilization. The analysis revealed that traditional villages exhibit a pronounced clustered spatial pattern with significant regional heterogeneity. Three principal hotspots—Anyang-Hebi in northern Henan, Pingdingshan in central Henan, and Xinyang in southern Henan—emerged as core concentration areas, corroborated by a nearest neighbor index (R = 0.6017, P = 0) and the imbalance index (S = 0.74). In contrast, western Henan displayed a relatively uniform distribution, reflecting its role as a transitional terrain zone, whereas eastern Henan showed sparse settlement, shaped by historical Yellow River flooding and significant modern urbanization impacts.

The spatiotemporal evolution of traditional villages in Henan Province exhibits a distinct directional transition, shifting from a “Southwest-Northeast” trajectory during the pre-Qin through Tang-Song-Yuan periods to a “Northwest-Southeast” orientation from the Ming-Qing to post-Qing eras. This transformation reflects the adaptive responses of rural settlements to historical dynamics, driven by advances in agricultural technology, such as the introduction of new crops and the construction of water conservancy infrastructure, along with changes in political status and migration patterns.

The spatial configuration of villages results from the synergistic interaction of multiple factors and pronounced spatial spillover effects. Descriptively, villages are concentrated at elevations of 100–300 m and in areas with GDP below 2,500 yuan/km^2^. However, after spatial autocorrelation was controlled for in the SLM, elevation and GDP were not statistically significant as independent predictors, suggesting that village distribution is shaped more by combined effects and spatial spillover than by any single variable alone. Hydrological proximity and distances of 10–20 kilometers from county-level cities further interact with these factors, collectively shaping the “moderate terrain + low urbanization” niche as the primary preservation zone. A pronounced spatial spillover (SLM β = 0.900, P < 0.01) enhances village agglomeration, reinforcing the formation of core settlement clusters.

In practical terms, these findings provide actionable guidance for protecting traditional villages and promoting rural revitalization. Targeted strategies should be applied to the three identified hotspots to maintain the synergy between natural environments and cultural heritage preservation. Cluster-based protection that focuses on contiguous village areas proves more effective than strategies focusing solely on individual sites, because it leverages spatial spillover effects to enhance conservation efficiency.

This study has several limitations. The sample includes only officially recognized surviving villages. The analysis is conducted at a macro scale, and cultural factors, such as historical administrative divisions and intangible heritage, are not quantitatively integrated.

Future research could integrate archeological and historical data to account for vanished settlements, undertake micro-case studies to investigate village-specific dynamics, and include a broader range of socio-cultural variables to refine the mechanisms of multi-dimensional interactions. Despite these constraints, this study advances the understanding of traditional village dynamics within agricultural civilization regions and offers practical insights for heritage preservation and sustainable rural development in the Central Plains. Conceptually, the study shows that traditional village persistence in the Central Plains is best explained not by a single factor, but by the joint effects of environmental adaptation, historical path dependence, and uneven urbanization. Henan therefore serves not only as a provincial case, but also as a representative example of how rural cultural landscapes survive and reorganize under long-term settlement evolution and contemporary development pressure.

## Supporting information

S1 Appendix Raw dataset of traditional villages in Henan Province. This Excel file contains the basic attributes of 1,035 traditional villages, including serial number, city, county/township, village name, batch, protection level, longitude, latitude, historical period, elevation, river distance, GDP, and county government distance.
(XLSX)

S2 Appendix Sensitivity analysis of kernel density estimation (KDE) bandwidth for traditional villages. This figure presents KDE results using candidate bandwidths of 30 km, 50 km, 70 km, and 90 km, supporting the selection of the optimal bandwidth of 50 km for the main analysis.
(TIF)

S3 Appendix GIS source files for the spatial analysis of traditional villages. This compressed file contains project files and intermediate layers for all spatial analyses, including maps for Figs 1, 3–8, and S2 Appendix, allowing full reproducibility of the spatial results.
(RAR)
